# Sociodemographic and clinical characteristics of patients with long-term remission with buprenorphine / naloxone in opiate use disorder

**DOI:** 10.1192/j.eurpsy.2021.2176

**Published:** 2021-08-13

**Authors:** Y. Kahya, A. Erdogan

**Affiliations:** Psychiatry, Akdeniz University Hospital, Antalya, Turkey

**Keywords:** opiate, remission, naloxone, buprenorphine

## Abstract

**Introduction:**

Buprenorphine/Naloxone (BP/NLX) is an effective drug combination used in long-term maintenance therapy in opiate use disorder (OUD). In some studies, abstinence over 180 days was defined as long-term remission (1).

**Objectives:**

The aim of this study is to determine the sociodemographic and clinical characteristics of patients in long-term remission with BP/NLX.

**Methods:**

In this study, 30 patients who were followed up with OUD at Akdeniz University Addiction Center and were in remission with BP/NLX for at least 180 days were evaluated retrospectively.

**Results:**

Sociodemographic and clinical characteristics are summarized in table 1.

**Figure:**
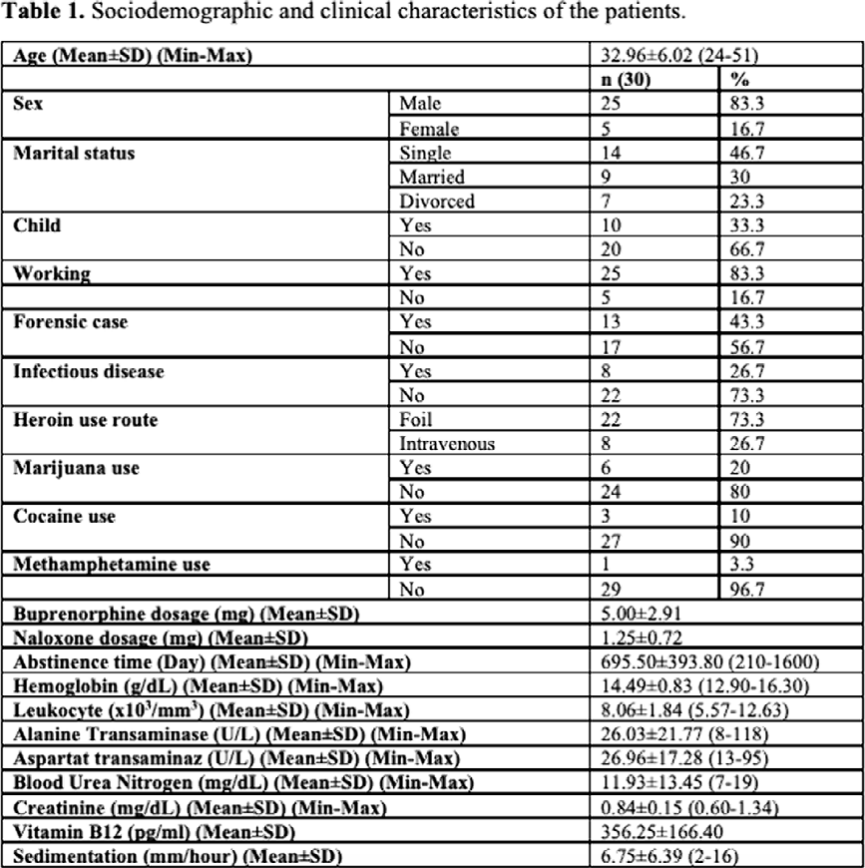

**Conclusions:**

OUD is associated with lower quality of life and employment rate (2). In our study, the rate of working in a regular job is high. It can be concluded that prolonged remission improves functionality in patients. Although patients are in remission for a long time in terms of opiate use, 20% of patients continue to use cannabis and 10% continue to use cocaine. In a study, there was no difference in productivity and quality of life between BP users with and without current cannabis use. Continued use of cannabis by patients may be related to this condition. However, cannabis use increases many mental illnesses, especially psychosis (4). In patients in remission with BP/NLX, studies should also be carried out to avoid other substances than opiates. In our study, in accordance with the literature (5), no negative effects on kidney and liver functions were observed with long-term BP/NLX treatment. BP/NLX appears to be safe in prolonged use.

**Disclosure:**

No significant relationships.

